# Metonymy Processing in Chinese: A Linguistic Context-Sensitive Eye-Tracking Preliminary Study

**DOI:** 10.3389/fpsyg.2022.916854

**Published:** 2022-07-27

**Authors:** Xianglan Chen, Hulin Ren, XiaoYing Yan

**Affiliations:** ^1^Center for the Cognitive Science of Language, Beijing Language and Culture University, Beijing, China; ^2^School of Foreign Studies, University of Science and Technology, Beijing, China

**Keywords:** metonymy processing, eye tracking, spillover contextual information, embodied cognition, preceding contextual information

## Abstract

Current cognitively oriented research on metaphor proposes that understanding metaphorical expressions is a process of building embodied simulations, which are constrained by past and present bodily experiences. However, it has also been shown that metaphor processing is also constrained by the linguistic context but, to our knowledge, there is no comparable work in the domain of metonymy. As an initial attempt to fill this gap, the present study uses eye-tracking experimentation to explore this aspect of Chinese metonymy processing. It complements previous work on how the length of preceding linguistic context influences metonymic processing by focusing on: (1) the contextual information of both the preceding target words; (2) the immediate spillover after the target words; and (3) whether the logical relationship between the preceding contextual information and the target word is strong or weak (a 2 × 2 between-subject experiment with target words of literal/metonymy and logic of strong/weak). Results show that readers take longer to arrive at a literal interpretation than at a metonymic one when the preceding information is in a weak logic relationship with target words, although this disparity can disappear when the logic is strong. Another finding is that both the preceding and the spillover contextual information contribute to metonymy processing when the spillover information does more to the metonymy than it does to the literal meaning. This study further complements cognitive and pragmatic approaches to metonymy, which are centered on its conceptual nature and its role in interpretation, by drawing attention to how the components of sentences contribute to the metonymic processing of target words. Based on an experiment, a contextual model of Chinese metonymy processing is proposed.

## Introduction

In a recent cognitive-linguistic account, metonymy is described as a domain-internal conceptual mapping based on expansion or reduction cognitive operations which combine with a formal substitution operation (which is not exclusive of metonymy; *cf.* Ruiz de Mendoza, 2017; see also Ruiz de Mendoza Ibáñez, 2021, for an overview of positions). Ruiz de Mendoza Ibáñez and Pérez Hernández (2001) have further proposed the term *high-level metonymy* to refer to metonymies where both the source and target domains are abstract domains. An example is GENERIC FOR SPECIFIC (e.g., *What is that building*?, meaning the identity of the building, say, the Royal Palace). These and other cognitive-linguistic studies on metonymy (*cf.* Blanco Carrión et al., 2018) focus on its conceptual composition, its boundaries with other figures of speech (especially metaphor) and its interaction with other metonymies and with metaphor. Studies based on inferential pragmatics deal with the interpretive aspects of metonymy sometimes both in cognitive and communicative terms (e.g., Wilson and Carston, 2007; Jodłowiec and Piskorska, 2015). However, such concept-based linguistically-oriented proposals, no matter how developed, do not clearly take into account the influence of the preceding or following linguistic context on the target words. The situation is only slightly different in psycholinguistic research, which in addressing different aspects of metonymic processing, has incidentally touched on contextual factors. For example, anaphoric inference in figurative referential description was discussed by Gibbs (1990), who found that subjects were faster at reinstating the antecedents for literal referential descriptions than for metaphoric and metonymic descriptions and that people understood metaphoric referential descriptions more easily than they did metonymic descriptions. Duffy and Rayner (1990) proposed the influence of distance on figurative description, suggesting that the distance to the figurative description also affects interpretation. Frisson and Pickering (1999, 2001) used the literal context and the metonymic context to find that processing unfamiliar metonymies was more difficult than processing familiar ones. McElree et al. (2006) reported on an experiment that examined the processing of standard metonymies (e.g., *The gentleman read Dickens*) and logical metonymies (e.g., *The gentleman began Dickens*), which they contrasted with control expressions with a conventional interpretation (*The gentleman met Dickens*). Eye movement measures during the reading process indicated that logical metonymies were more costly to interpret. Experiments that have used preceding context that affects metonymic interpretation (e.g., Frisson and Pickering, 2007; Piñango et al., 2017; Chen and Li, 2019) have suggested that, only when the preceding context is adequate in terms of support for underspecification and the resolution of interpretive variability, is metonymic processing almost the same as the literal interpretation.

In the present study, we wondered if the context of preceding or following information could also affect metonymic processing. A few words of caution are necessary before we proceed to further contextualize and present our experimental work. First, such work is based on Chinese subjects. This means that our claims are valid only for the understanding of processing by native speakers of this language. To place this study fully within the context of others, based on English, it would be necessary to carry out a contrastive study, which is not our goal. However, the study we offer can be replicated for other languages in future work. Second, metonymy is a complex phenomenon. Scholars have distinguished lexical metonymy from predicative, predicational, and even illocutionary metonymy (Ruiz de Mendoza Ibáñez and Galera, 2014, p: 66; see also Panther, 2005, and Panther and Thornburg, 2018). Also, as noted above, even within the same level of metonymic activity (e.g., lexical) some metonymies involve more conceptual complexity than others (which is the case of metonymic chains; Barcelona, 2005; Brdar-Szabó and Brdar, 2011; *cf.* Ruiz de Mendoza Ibáñez and Galera, 2014, p: 117) and metaphor has long been found to interact with metonymy (Goossens, 1990). The present empirical study is an initial approximation to such a complex phenomenon. For this reason, we have chosen to discuss an indisputable and simple example of metonymy, ‘scalpel for surgeon’ (INSTRUMENT FOR USER). Again, future work should consider more complex analytical situations.

## Literature Review

### The Four Models of Figurative Language Processing

Since the beginning of figurative language research, many psycholinguistic studies (e.g., Inhoff et al., 1984; Gibbs, 1986, 1990; Keysar, 1989; Cronk et al., 1993; Onishi and Murphy, 1993; Schraw, 1995) have compared the temporal sequences involved in accessing the literal and figurative meanings of non-idiomatic (*He is an icebox*) and idiomatic metaphor (*He blew his stack*). Based on these comparisons, four models of figurative language processing have been proposed: the literal first model (Searle, 1979; Grice, 1989), the figurative first model (Gibbs, 1980; Estill and Kemper, 1982; Gibbs and Gonzales, 1985), the parallel model (Paivio, 1979; Glucksberg, 1991), and the underspecification model (Frisson and Pickering, 1999, 2001).

The literal first model is also known as the standard pragmatic view (Grice, 1975; Searle, 1979) or the indirect access model (Lowder and Gordon, 2013). In this model, access to the non-figurative meaning of a trope always precedes access to its figurative interpretation, which is adopted only when the literal meaning is rejected in relation to the context of the sentence. Although this model was supported by some early empirical studies (e.g., Janus and Bever, 1985), it was also challenged by the fact that, if sufficient context is provided, the processor accesses the figurative meaning first (Gibbs, 1980). Because of this, researchers put forward the figurative-first or direct access model (Gibbs and Gonzales, 1985), where subjects begins with a figurative interpretation when processing what appears to be figurative language but turn to the literal meaning when a figurative interpretation cannot be found or when the figurative interpretation is incongruous in the context of the sentence. Evidence for this model comes from psycholinguistic studies that have suggested that the figurative interpretation of an idiom is accessed before the non-figurative interpretation (Gibbs, 1986). The figurative-first model was later challenged by proponents of the parallel model (Glucksberg, 1991; Cacciari and Glucksberg, 1994), which asserts that, if both literal and figurative interpretations are appropriate in a context, neither is accessed prior to the other (Gerring and Healy, 1983; Myers et al., 1987). Similarly, Gerrig (1989) formulated the concurrent processing model, which maintains that the selection and creation of meaning in the processing of figurative language operate simultaneously. These three models of figurative language processing depend on a dichotomy between figurative and non-figurative meanings to compare the chronology of their access. Using a different focus, Frisson and Pickering (1999, 2001) proposed the underspecification model, which provides a more general explanation by dividing the processing of figurative description into two stages: (1) a schematic stage, in which an underspecified, schematic representation of the meaning of a figurative description is developed; and (2) a home-in stage, in which a specific, appropriate meaning of the figurative description is determined with the aid of contextual and lexical information. The present study explores metonymic processing in Chinese and establishes its own model, which is cognitive and contextual in nature, thus adding significantly to the pool of previous studies.

The embodiment hypothesis has a great influence on the understanding of figurative language, especially for metaphor interpretation. Challenging the view of the disembodied mind, the embodiment hypothesis holds that our conceptual system is based on and shaped by our bodily experience and the interactions with the environment (Lakoff and Johnson, 1999; Gibbs, 2006a). For example, while understanding metaphor, “people may create embodied simulations of speakers’ messages that involve moment-by-moment ‘what must it be like’ processes which make use of ongoing tactile-kinesthetic experiences.” (Gibbs, 2006b, p: 455). Nowadays, the notion of embodiment has been applied to explain various kinds of figurative language, such as metaphor, metonymy, and irony (Filik et al., 2015). The empirical findings in the present paper are consistent with the embodied view of figurative language (see “Discussion and Conclusion”).

### Eye-Tracking Studies and the Processing of Metonymy

Not many studies have addressed the processing of metonymy. Among them, there is work based on processing times (Gibbs, 1990), on offline comprehension tasks (Slabakova et al., 2013), self-pace reading and probe recognition (Zarcone et al., 2014), and reaction times for sensicality judgments (Bott et al., 2016). There are also a few studies based on eye-tracking technology (Frisson and Pickering, 1999; Lowder and Gordon, 2013; Chen and Li, 2019). There follows a brief overview of some of this latter work.

The subjects in Pickering and Traxler (1998) read a context sentence followed by a syntactically ambiguous target sentence that included a metonymy. The study showed that, although unfamiliar metonymic expressions were not interpreted as quickly as literal expressions, people could access them rapidly when they were given appropriate contextual information. Frisson and Pickering (1999) addressed this issue by comparing the processing of conventional and novel metonymies in their figurative and literal contexts using two eye-tracking experiments. They found that the processing of novel metonymies was more difficult than processing conventional metonymies in figurative contexts and that, compared with the literal context, the figurative context had a weak effect on people’s ability to understand familiar metonymies. Their study highlighted the role of sentence context (or co-text) in understanding metonymies, especially unfamiliar ones. Frisson (2009) suggested that the underspecification model (Frisson and Pickering, 1999) prevailed in the processing of familiar metonymies in that readers begin their processing with a single, underspecified meaning instead of choosing between literal and figurative meanings. In a later study, Lowder and Gordon (2013) used eye-tracking experiments to determine the role of sentence structure in the processing of metonymy (Lowder and Gordon, 2013). The results indicated that the meanings of place-for-institution metonymies (e.g., *the White House* to refer to the American president and his advisers) were accessed more slowly than those metonymies which were the argument of a verb. The study also showed that, in the argument position, the metonymic expression required more time to process than ordinary nouns that refer to a person. These findings demonstrate that syntactic structure can modulate the processing of figurative language. Frisson and Pickering (2007) suggested that an adequate sentential context (or co-text) could facilitate the processing of a novel metonymy. Chen and Li (2019) also experimented with how the different lengths of preceding information affected the processing of metonymy in Chinese. The results showed that, with short sentence contexts, readers took longer to produce a metonymic interpretation than a literal one for unfamiliar metonymies. However, the processing disparity between metonymic comprehension and literal comprehension disappeared when more extensive and supporting information was available in the preceding sentence context.

In this context of empirical work, the present study takes up a still unexplored topic. It deals with how different levels of logic (strong/weak) work with the processing of literal/metonymic descriptions in Mandarin Chinese given contextual information from sentences of the same length. To be clear, a strong logic is one in which the information preceding the target words provides cause-effect evidence for its conclusion. For example, in the sentence *bingren taiduo, na ba* shoushudao *queshi shiyong tai pinfan le* (lit. Patients too many, that CL ***scalpel*** indeed use too frequently, where CL stands for classifier; ‘Since there are too many patients, that scalpel is used too frequently indeed’), the surgeon needs to operate frequently **because of** the excess of patients. Our hypothesis is that it will be easier to understand the target words under strong logic conditions. By contrast, a weak logic is one that information before and after the target words shows no cause-effect relation. We hypothesize that it will be harder to understand the target words under such conditions. For example, in the sentence *Shiyanshi li na ba* shoushudao *queshi shiyong tai pinfan le* (laboratory in, that CL *scalpel* indeed use too frequently ASP, ‘In that laboratory that scalpel is used too frequently’), the relationship between the text preceding *shoushudao* (‘scalpel’) and the text following it is not a cause-effect one.

To address this goal, we put forward the following research questions:

1. How does the preceding context facilitate the literal and metonymic description of the part-whole person metonymy type in Mandarin Chinese?
2. How does the level of logic (strong/leak) affect the relationship between the preceding context and the interpretation of the metonymic description of this metonymy type?
3. How does spillover text work in processing metonymy of this type?

## Materials and Methods

### Participants

Forty students (15 males and 25 females, mean age 21.6, age range 18–27) majoring in linguistics at Peking University were recruited for the experiment. All were native speakers of Chinese and all had normal eyesight.

### Materials

Eighty sets of sentences were constructed in Chinese. Each set had four types of conditions: (a) the literal-weak logic condition, in which the target word of the literal sense is used with a weak-logic preceding context; (b) the literal-strong condition, in which the target word is used in a literal sense with a strong-logic preceding context; (c) the metonymic-weak condition, in which the target word is used in a metonymic sense with aa weak-logic preceding context; and (d) the metonymic-strong condition, in which the target word is used in the metonymic sense with a strong-logic preceding context. The following set of sentences, with target words in bold, is an example of the material used in the experiment.

1 a. *Shiyanshi li, na ba **shoushudao** queshi shiyong tai pinfan le*.

laboratory in, that CL **scalpel** indeed use too frequently ASP.

‘That scalpel in the laboratory was indeed used too frequently.’

b. *bingren taiduo，na ba **shoushudao** queshi shiyong tai pinfan le*.

patients too many, that CL **scalpel** indeed use too frequently ASP.

‘Since there are so many patients, that scalpel was indeed used too frequently.’

c. *shiyanshi li, na wei shoushudao queshi zuotian mei lai zhudao.*

laboratory in, that CL **scalpel** indeed yesterday neg. Come operate.

‘That scalpel did not come to perform the operation in the laboratory yesterday.’

d*. chuchai zai wai, na wei shoushudao queshi zuotian mei lai zhudao.*

on business in outside, that CL **scalpel** indeed yesterday neg. Come operate.

‘Because (he) was on a business trip, that scalpel did not come to perform the operation yesterday.’

In Chinese, people use *shoushudao* (scalpel) either literally to refer to a tool, as in (a) and (b) or metonymically to refer to a doctor, as in (c) and (d). In 1(a) and 1(b), the word *ba* is used as a classifier to disambiguate the literal and metonymic meanings of *shoushudao*, as *ba* is an indicator of an object rather than a person. In 1(c) and (d), the word *wei* is used as a classifier to disambiguate the two possible senses, as *wei* is used only to modify people (rather than things). However, different levels of logic appear between the preceding context and the target words.

### Pretest for the Experimental Material

To assess the appropriateness of our 80 sets of sentences before the pretest, 12 native Chinese speakers with PhD and MR degrees in psycholinguistics or linguistics were hired to screen out inappropriate sentences based primarily on intuition and to choose familiar metonymy and ensure literal or metonymic descriptions.

Non-experimental metonymy-identification procedures have been discussed in the recent literature on metonymy. Littlemore (2015, p: 127) puts forward a procedure adapted from the well-known metaphor-identification procedure in Steen (2007; see also Zibin, 2021, p: 33). In application of this procedure to one of our examples, *At school, this long-haired often travels with me*, the researcher first identifies metonymy-related words (‘long-haired’), secondly propositions (‘person who has long hair’), thirdly denoted entities (long hair and person), and finally, the researcher makes conceptual links between the denoted concepts and their associated conceptual domains: the fact that the hair is part of a person suggests a part-for-whole metonymic configuration where the long hair stands for the person characterized by having long hair. This procedure is systematic, but it relies on the analyst’s knowledge, which can be considered subjective. In order to endow the identification of metonymies with intersubjectivity, which is akin to objectivity if supported by a reliable statistical procedure, we complemented the analyst’s identification process with a questionnaire-based protocol described below.

Sixteen sets of sentences that were grammatically correct but pragmatically unreasonable were removed from the experimental material. Then the remaining 64 sets of sentences were randomized into four lists by means of a Latin square design for post-questionnaire using a five-point Likert scale. Two pretests were conducted to assess the sentences’ acceptability in terms of effective selection of metonymies and the relevance of the preceding adverbial to the target word by means of a questionnaire that used a five-point Likert scale. The relevance test was performed to ensure that the distinction between the condition of weak logical contextual information and that of strong logical contextual information was clear. The results of the pretest on the acceptability of the experimental metonymic material showed a similar main effect of sense [*F_2_* (1,63) = 12.537, *p* = 0.001, *η*^2^ = 0.166]. In the relevance test, the material that preceded the comma was perceived as being more logical in its relationship to the target word under the condition of strong logical information than it was under the condition of weak logical information (*M* = 4.071 versus 3.176, *p* = 0.000), as expected. In summary, the data confirmed that the selection of metonymies was effective and the manipulation of the relationship between the metonymies and the logical context was successful.

Finally, the 64 sets of sentences were divided into four lists using a Latin square design, with each set consisting of four conditions. To avoid the effect of stimulus predictability, we added 76 sentences to each list as fillers, and pseudo-randomization was applied to all lists.

### Equipment and Procedure

We employed the eye tracker EyeLink 1,000, which uses infrared video-based tracking technology to sample the subjects’ eyes at 1,000 Hz. The stimuli were programmed to be displayed on a computer screen about 50 centimeters away from the viewers. Participants took the test at a Peking University laboratory. Written instructions were distributed asking the participants not to move their heads or blink excessively during the experiment. Then the eye tracker was adjusted and calibrated, after which 10 practice items were presented, followed by a one-minute break before the real experiment began. Participants were asked to look at a black dot in the middle of the screen, followed by a crosshair at the exact point at which the first Chinese character in each sentence would appear. If the participant’s eyes did not fix on the crosshair within a certain period of time, the black dot reappeared, as the test sentence would not appear until the participant’s eyes were fixed on the crosshair. The participants were then asked to read the sentences and press a key to trigger a yes-no response to a question that checked their understanding of the test sentences. To keep a balance across the conditions, half of the comprehension questions required a “yes” answer, and the other half required a “no” answer. After answering the comprehension questions, participants moved on to the next trial. The entire experiment lasted approximately 50 min, divided into two sessions with a predetermined break in the middle. If the participant became tired or blinked too much during the experiment, the experiment was paused to allow the participant to rest.

## Findings

Eye-fixations that were shorter than 80 msec were removed from the data, as the participants could not have processed any information during such a short time (Henderson et al., 1989). Ninety-two trials (3.5%) in which the participants provided incorrect answers to the comprehension questions were removed from the study.

In the next phase of the test, four areas of interest (AOIs) were established in every sentence for observation: the adverbial in front of the comma, the classifier, the target word, and the spillover region. For the purpose of making clear the processing of each of the areas before and after the target word, six eye-movement measures were used in each of the four AOIs: *the duration of the first fixation* (the length of the first fixation on the current AOI); *the first-run dwell time* (the length of all fixations on the current AOI in the first run); *the second-run dwell time* (the length of all fixations on the current AOI in the second run); *regression in count* (the number of times the participant looked back at an AOI from a point later in the sentence); *duration of the regression path* (the time from when the first fixation on the AOI started to the end of the last fixation before leaving the AOI, which includes not only *first-run dwell time* of the region but also the rereading time of the previous regions); and *dwell time* (the sum of time spent on an AOI).

## Results

A general linear model repeated measures analysis was applied to analyze the eye-movement data. The results of ANOVAs are reported based on both the means for each subject across items (*F_1_*) and the means for each item across subjects (*F_2_*). Results from each critical region are discussed separately to represent the nature of the observed effects.

### Target-Word Region

The target-word region refers to the literal or metonymic description in the sentence, which is the core part of the experiment. Table 1 shows the mean and standard errors of the participants’ six eye-movement measures.

**Table 1:** Mean reading times and standard errors for the target word.

**Measures**	**Condition**			
	**Literal-less**	**Literal-more**	**Metonymic-less**	**Metonymic-more**
Duration of first fixation	225.60(5.77)	214.87(4.68)	213.38(5.86)	220.40(5.60)
First-run dwell time	275.76(9.89)	262.79(9.33)	265.52(9.82)	273.85(10.37)
Regression in count	0.45(0.041)	0.38(0.041)	0.48(0.048)	0.41(0.045)
Duration of the regression path	320.91(14.17)	308.61(11.93)	304.81(16.31)	315.71(13.91)
Second-run dwell time	261.61(11.46)	256.18(11.58)	263.14(18.41)	251.74(10.13)
Dwell time	407.45(19.65)	369.54(19.77)	405.83(16.72)	397.61(18.17)

As Table 1 shows, two main effects on two eye-movement measures: regression in count and dwell time. Regression in count shows that a preceding context with weak logical information caused the target words to receive more regressions from a later area [*F_1_*(1,39) = 15.068, *p* = 0.000, *η*^2^ = 0.279; *F_2_*(1,63) = 11.648, *p* = 0.001, *η*^2^ = 0.156], while dwell time on target words was longer under the condition of weak logic than under that of strong logical information [*F_1_*(1,39) = 5.591, *p* = 0.023, *η*^2^ = 0.125; *F_2_*(1,63) = 4.809, *p* = 0.032, *η*^2^ = 0.071]. Both the item analysis and the subject analysis reveal a significant interaction in the duration of the first-run fixation [*F_1_*(1,39) = 9.116, *p* = 0.004, *η*^2^ = 0.189; *F_2_*(1,63) = 7.587, *p* = 0.008, *η*^2^ = 0.107]. Further analysis of this simple effect demonstrates that the duration of the first-run fixation was longer for target words with literal sense than it was for target words with metonymic sense when there was less information in the preceding context [*F_1_*(39) = 6.189, *p* = 0.017; *F_2_*(63) = 4.207, *p* = 0.047]. Meanwhile, when the target words required literal interpretation, they saw longer duration of fixation in the first run under the condition of weak logic than under the condition of strong logic [*F_1_*(39) = 6.256, *p* = 0.017; *F_2_*(63) = 8.109, *p* = 0.006]. This result is supportive of the main effect mentioned above.

### Adverbial Region

When we explore the adverbial region, which works as part of the preceding context, it is helpful to know the degree to which the preceding context functions in the processing of the key words. Table 2 shows the mean of participants’ eye-movement data regarding the adverbial region.

**Table 2:** Mean reading time for the adverbial region.

**Measures**	**Condition**			
	**Literal-less**	**Literal-more**	**Metonymic-less**	**Metonymic-more**
Duration of the first fixation	278.19(4.14)	288.22(5.44)	280.93(4.57)	279.12(4.94)
First-run dwell time	458.11(15.20)	558.90(23.45)	457.76(15.64)	520.97(19.06)
Regression in count	0.37(0.047)	0.50(0.052)	0.35(0.048)	0.44(0.053)
Duration of the regression path	459.65(15.06)	567.38(23.54)	459.66(16.33)	523.90(19.18)
Dwell time	588.92(29.37)	739.76(35.63)	587.30(28.29)	667.73(31.43)

As Table 2 shows, for the difference between literal and metonymic description, there is a significant main effect of sense in three measures [first-run dwell time: *F_1_*(1,39) = 6.116, *p* = 0.018, *η*^2^ = 0.136; *F_2_*(1,63) = 3.517, *p* = 0.065, *η*^2^ = 0.053; duration of the regression path: *F_1_*(1,39) = 7.067, *p* = 0.011, *η*^2^ = 0.153; *F_2_*(1,63) = 4.247, *p* = 0.043, *η*^2^ = 0.063; and dwell time: *F_1_*(1,39) = 10.738, *p* = 0.002, *η*^2^ = 0.216; *F_2_*(1,63) = 3.976, *p* = 0.050, *η*^2^ = 0.059], suggesting that participants spent more time reading the adverbial region when they were in the literal condition than when they were in the metonymy condition. This result is supported by another two measures—the duration of fixation duration and regression in count—which showed a marginally significant main effect of sense, although only in the analysis of the subject [duration of the first fixation: *F_1_*(1,39) = 3.277*p* = 0.078, *η*^2^ = 0.078; *F_2_*(1,63) = 1.310, *p* = 0.257, *η*^2^ = 0.020; regression in count: *F_1_*(1,39) = 2.855, *p* = 0.099, *η*^2^ = 0.068; *F_2_*(1,63) = 1.217, *p* = 0.274, *η*^2^ = 0.019].

The preceding context had a significant main effect on the same three measures [first-run dwell time: *F_1_*(1,39) = 82.789, *p* = 0.000, *η*^2^ = 0.680; *F_2_*(1,63) = 41.764, *p* = 0.000, *η*^2^ = 0.399; duration of regression path: *F_1_*(1,39) = 82.871, *p* = 0.000, *η*^2^ = 0.680; *F_2_*(1,63) = 47.543, *p* = 0.000, *η*^2^ = 0.430; dwell time: *F_1_*(1,39) = 99.393, *p* = 0.000, *η*^2^ = 0.718; *F_2_*(1,63) = 34.573, *p* = 0.000, *η*^2^ = 0.354], as well as the regression count [*F_1_*(1,39) = 22.631, *p* = 0.000, *η*^2^ = 0.367; *F_2_*(1,63) = 15.810, *p* = 0.000, *η*^2^ = 0.201], revealing that participants spent more time reading the adverbial region when there was weak logic in the preceding information. Support for this result comes from two other measures: duration of the first fixation, which showed a marginally significant effect of preceding context only in the item analysis [*F_1_*(1,39) = 1.935, *p* = 0.172, *η*^2^ = 0.047; *F_2_*(1,63) = 2.964, *p* = 0.090, *η*^2^ = 0.045], and the second-run dwell time, which showed a significant effect of the preceding context only in the subject analysis [*F_1_*(1,39) = 5.587, *p* = 0.024, *η*^2^ = 0.138; *F_2_*(1,63) = 1.935, *p* = 0.169, *η*^2^ = 0.032].

The subject analysis and the item analysis also have a significant interaction effect on four of the six measures [duration of the first fixation: *F_1_*(1,39) = 4.457, *p* = 0.041, *η*^2^ = 0.103; *F_2_*(1,63) = 3.884, *p* = 0.053, *η*^2^ = 0.058; first-run dwell time: *F_1_*(1,39) = 12.951, *p* = 0.001, *η*^2^ = 0.249; *F_2_*(1,63) = 4.560, *p* = 0.037, *η*^2^ = 0.067; duration of the regression path: *F_1_*(1,39) = 16.420, *p* = 0.000, *η*^2^ = 0.296; *F_2_*(1,63) = 6.062, *p* = 0.017, *η*^2^ = 0.088; dwell time: *F_1_*(1,39) = 12.425, *p* = 0.001, *η*^2^ = 0.242; *F_2_*(1,63) = 4.253, *p* = 0.043, *η*^2^ = 0.063]. Additional analysis of these simple effects shows no difference in the time participants spent reading adverbials between the metonymic and literal sentences when weak logic was provided [i.e., condition (a) vs. condition (c); duration of first fixation: *F_1_*(39) = 0.596, *p* = 0.445; *F_2_*(63) = 0.390, *p* = 0.534; first-run dwell time: *F_1_*(39) = 0.016, *p* = 0.939; *F_2_*(63) = 0.006, *p* = 0.900; duration of the regression path: *F_1_*(39) = 0.035, *p* = 0.852; *F_2_*(63) = 0.020, *p* = 0.888; dwell time: *F_1_*(39) = 0.029, *p* = 0.865; *F_2_*(63) = 0.010, *p* = 0.920]. However, the difference between the literal and the metonymic sentences was significant when strong logical information was provided in the preceding context [i.e., condition (b) vs. condition (d); duration of the first fixation: *F_1_*(39) = 7.292, *p* = 0.010; *F_2_*(63) = 4.574, *p* = 0.036; first-run dwell time: *F_1_*(39) = 15.1, *p* = 0.017; *F_2_*(63) = 5.991, *p* = 0.000; duration of the regression path: *F_1_*(39) = 18.557, *p* = 0.000; *F_2_*(63) = 7.326, *p* = 0.009; dwell time: *F_1_*(39) = 19.848, *p* = 0.000; *F_2_*(63) = 6.483, *p* = 0.013]. In other words, when the context was strong logical, participants spent more time on the adverbial region when the target words required a literal interpretation than they did when the target words required a metonymic interpretation. In addition, when a literal interpretation of the target words was required, condition (a) and (b) showed a significant difference [duration of the first fixation: *F_1_*(39) = 5.545, *p* = 0.024; *F_2_*(63) = 5.729, *p* = 0.020; first-run dwell time: *F_1_*(39) = 67.005, *p* = 0.000; *F_2_*(63) = 34.827, *p* = 0.000; duration of the regression path: *F_1_*(39) = 72.718, *p* = 0.000; *F_2_*(63) = 40.996, *p* = 0.020; dwell time: *F_1_*(39) = 71.647, *p* = 0.000; *F_2_*(63) = 28.694, *p* = 0.002]: that is, participants spent more time on the adverbial region when strong logical information was provided in the preceding context when literal interpretation was required. The same result was found in three measures for the sentences that required metonymic interpretations [i.e., conditions (c) and (d); first-run dwell time: *F_1_*(39) = 53.026, *p* = 0.000; *F_2_*(63) = 23.992, *p* = 0.020; duration of the regression path: *F_1_*(39) = 48.116, *p* = 0.000; *F_2_*(63) = 23.930, *p* = 0.020; dwell time: *F_1_*(39) = 47.784, *p* = 0.000; *F_2_*(63) = 10.879, *p* = 0.002].

### Classifier Region

We performed an analysis to determine the contribution of the classifier region to metonymic processing key words. Table 3 shows the average time the participants spent reading the classifier region (including a demonstrative and a classifier) under each condition for the eye movements measures.

**Table 3:** Reading times of the classifier region.

**Measures**	**Condition**			
	**Literal-less**	**Literal-more**	**Metonymic-less**	**Metonymic-more**
Duration of the first fixation	222.26(8.84)	228.180(8.80)	223.86(7.19)	230.79(6.95)
First-run dwell time	244.380(13.29)	249.39(13.27)	241.94(10.83)	249.99(9.97)
Regression in count	0.49(0.048)	0.4(0.048)	0.48(0.049)	0.44 (0.066)
Duration of the regression path	268.33(14.56)	287.48(14.71)	260.31(10.49)	281.58(11.87)
Dwell time	186.73(20.99)	207.40(22.00)	198.97(21.24)	193.17(20.17)

As Table 3 shows, the preceding context has a significant main effect on two eye-movement measures: regression in count [*F_1_*(1,39) = 3.778, *p* = 0.059, *η*^2^ = 0.090; *F_2_*(1,63) = 5.624, *p* = 0.021, *η*^2^ = 0.083] and duration of the regression path [*F_1_*(1,39) = 10.137, *p* = 0.003, *η*^2^ = 0.211; *F_2_*(1,63) = 3.629, *p* = 0.061, *η*^2^ = 0.055], so the classifier region attracted more regression time from the later areas of target words when strong, instead of weak, logic was provided. The subject analysis of two other measures supported the result too. The duration of the first fixation and the first-run dwell time also presented a significant or marginally significant main effect of the preceding context [duration of the first fixation: *F_1_*(1,39) = 5.538, *p* = 0.024, *η*^2^ = 0.127; *F_2_*(1,63) = 1.306, *p* = 0.258, *η*^2^ = 0.021; first-run dwell time: *F_1_*(1,39) = 2.899, *p* = 0.097, *η*^2^ = 0.071; *F_2_*(1,63) = 1.170, *p* = 0.283, *η*^2^ = 0.019], demonstrating that the classifier region received more attention initially, when strong logical information was in the preceding context.

### Spillover Region

The spillover region is explored to determine if this part of the sentence context contributes to the keywords of the metonymy. This examination is the first for this part of the context, but the Chinese language is not as linear as English, so further exploration is needed. Table 4 shows the means of the participants’ six eye-movement measures in the spillover region (with at least two Chinese characters immediately following the target word in each sentence) under the four conditions.

**Table 4:** Mean reading time of the spillover region.

**Measures**	**Condition**			
	**Literal-less**	**Literal-more**	**Metonymic-less**	**Metonymic-more**
Duration of the first fixation	219.48(4.78)	227.58(6.36)	231.20(6.30)	221.53(5.25)
First-run dwell time	242.78(7.66)	257.13(10.51)	262.25(9.97)	249.67(8.56)
Regression in count	0.30(0.031)	0.23(0.03)	0.29(0.035)	0.30(0.035)
Duration of the regression path	311.68(14.10)	317.20(16.01)	329.42(14.88)	330.098(16.40)
Second-run dwell time	237.26(8.71)	238.89(11.35)	241.13(11.95)	239.17(11.12)
Dwell time	296.57(16.86)	297.40(15.44)	327.59(17.45)	316.86(16.03)

Table 4 shows that the spillover region has a significant main effect on the dwell time [*F_1_*(1,39) = 7.462, *p* = 0.009, *η*^2^ = 0.161; *F_2_*(1,63) = 5.084, *p* = 0.028, *η*^2^ = 0.075], indicating that participants took less time to read the spillover region if the target words required a literal interpretation than when these words required a metonymic interpretation.

Meanwhile, two measures indicating early processing, duration of the first fixation and first-run dwell time, reveal a significant interaction effect [duration of the first fixation: *F_1_*(1,39) = 4.009, *p* = 0.052, *η*^2^ = 0.093; *F_2_*(1,63) = 5.435, *p* = 0.023, *η*^2^ = 0.081; first-run dwell time: *F_1_*(1,39) = 4.812, *p* = 0.034, *η*^2^ = 0.110; *F_2_*(1,63) = 5.646, *p* = 0.021, *η*^2^ = 0.083]. Further study of this simple effect reveals that, when the preceding context contains weak logical information, conditions (a) and (c) are significantly different [duration of the first fixation: *F_1_*(39) = 2.865, *p* = 0.098; *F_2_*(63) = 4.996, *p* = 0.029; first-run dwell time: *F_1_*(39) = 5.165, *p* = 0.029; *F_2_*(63) = 5.362, *p* = 0.024], which shows that the spillover region receives less reading time if a literal interpretation of the target words, rather than the metonymic interpretation, is required. However, if more information is included in the preceding context, the difference between the literal condition (b) and the metonymic condition (d) becomes less significant [duration of the first fixation: *F_1_*(39) = 1.800, *p* = 0.187; *F_2_*(63) = 1.566, *p* = 0.215; first-run dwell time: *F_1_*(39) = 1.340, *p* = 0.254; *F_2_*(63) = 1.333, *p* = 0.253]. In addition, when the target words require a literal interpretation, the difference in the time the participants spent reading the spillover region between the logical context conditions (a) and (b) was insignificant [duration of the first fixation: *F_1_*(39) = 1.874, *p* = 0.179; *F_2_*(63) = 3.139, *p* = 0.081; first-run dwell time: *F_1_*(39) = 3.092, *p* = 0.087; *F_2_*(63) = 5.362, *p* = 0.024]. However, when a metonymic interpretation was required, the difference between conditions (c) and (d), where the spillover region received a longer first fixation when weak logical information was provided in the preceding context, was marginally significant [*F_1_*(39) = 3.310, *p* = 0.077; *F_2_*(63) = 2.606, *p* = 0.112].

## Discussion and Conclusion

This study differs from other studies on metonymy in that it considers only the logical relationship between the preceding sentence context and the target words. Ortony et al. (1978) proposed that preceding text inspired readers to form a framework for understanding the figurative language, but the present study goes further to show how regions of both the preceding sentence context and the following sentence context (the spillover region) contribute to literal and metonymic processing.

**Figure 1:**
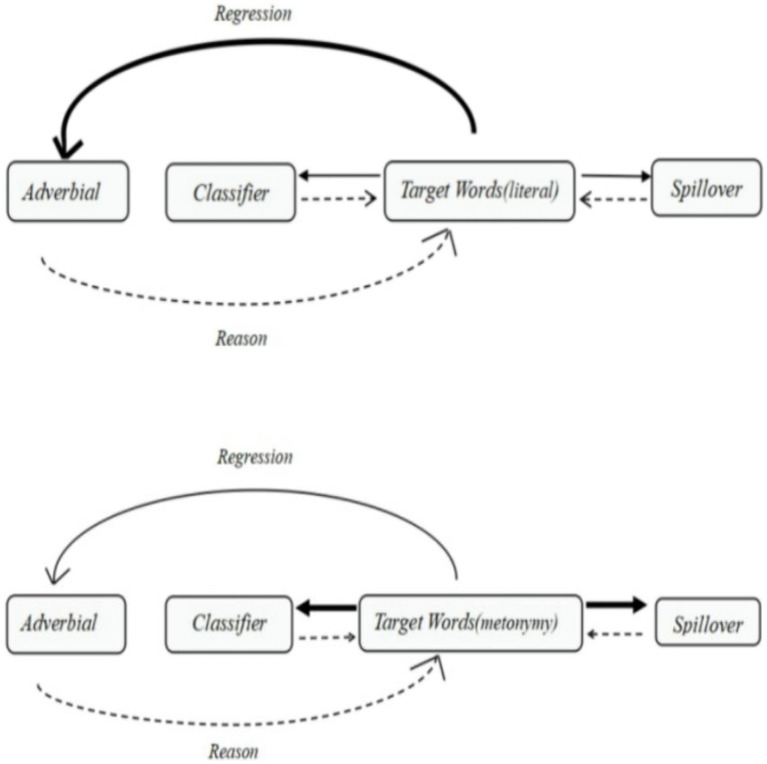
Cognitive-psychological model for Chinese metonymy as subject.

The results show that target words are processed faster when the preceding context contains strong, rather than weak, logic in relation to the key words, regardless of whether the target words require metonymic or literal interpretation. This outcome indicates that the meaning of the target word comes not only from the target word itself, but also from the reasoning from regression pass duration with regression counts. To be more exact, the time spent interpreting the two literal conditions [conditions (a) and (b)] is longer than that for the two metonymic conditions [conditions (c) and (d)]. The time spent in condition (a) was much longer than that in condition (b), suggesting that strong logical information in the preceding context (b) facilitates the processing of literal senses. Literally used target words in Mandarin Chinese were more sensitive to the preceding logical information, but the disparity in reading time between literal and metonymic meanings could be reduced if strong logical information were available in the preceding context. Metonymic target words depend more on the classifier than the logical information of the previous context, as illustrated in Figure 1.

As for the measure of the duration of the regression path in the adverbial region, the logic in the preceding context facilitates the processing of both the literal and metonymic target words, although the logic works more effectively in the literal than in the metonymic meaning. The dwell time offers the same support, although the classifier region and the spillover with the regression time contribute more to metonymy processing.

In this experiment, Frisson and Pickering’s (2001) under-specification model is most likely to provide an explanation for the processing of metonymy, where meaning is underspecified in that the initial interpretations are compatible with many senses instead of only a certain sense. Based on contextual information, readers can then refine the meaning to fit a particular sense. If the preceding context is helpful in determining the appropriate sense, readers may home in on the specific sense more rapidly. The experiment conducted here shows that metonymic processing requires more contribution from the classifier that precedes it and the spillover that follows it than does literal processing.

This study also suggests that people’s interpretation of metonymy partly involved creating an embodied simulation along with contextual information. Even though relationships are not physical entities that literally travel along physical paths, people nonetheless conceive of relationships in metaphorical ways, especially when prompted to do so by contextual information. This metonymic conceptualization is not purely abstract, but embodied in the sense that participants imagine themselves in the situation and experience. Of course, the study above used a specific sentence, and it is also unclear whether these findings generalize to other kinds of figurative language, including different types of metonymy (Gibbs, 2006b).

The findings of the study support the notion that familiar metonymy may result in faster processing. The results of the eye-tracking experiment also provide strong evidence for the contribution of the different regions to interpretation. Future research is needed to determine how the classifier (CL) contributes to processing the target words.

## Data Availability Statement

The original contributions presented in the study are included in the article/supplementary material, further inquiries can be directed to the corresponding author.

## Author Contributions

XC provides the idea and design and writes the draft paper. HR is in charge of the experiment. XY gets the data and does the analysis. All authors contributed to the article and approved the submitted version.

## Funding

We are grateful for the financial support provided by National Philosophy and Social Science Fund Program, number: 19BYY016 and the Project Funded by Ministry of Education, number: 19JHQ035 and University Project KCSZ202123.

## Conflict of Interest

The authors declare that the research was conducted in the absence of any commercial or financial relationships that could be construed as a potential conflict of interest.

## Publisher’s Note

All claims expressed in this article are solely those of the authors and do not necessarily represent those of their affiliated organizations, or those of the publisher, the editors and the reviewers. Any product that may be evaluated in this article, or claim that may be made by its manufacturer, is not guaranteed or endorsed by the publisher.

## References

[ref1] Barcelona A. (2005). “The multilevel operation of metonymy in grammar and discourse, with particular attention to metonymic chains,” in Cognitive Linguistics. Internal Dynamics and Interdisciplinary Interaction. eds. Ruiz de Mendoza F. J. Peña S. (Berlin & New York: Mouton de Gruyter), 313–352.

[ref2] Blanco Carrión O. Barcelona A. Pannain R. (Eds.) (2018). Conceptual Metonymy. Methodological, Theoretical, and Descriptive Issues. Amsterdam & Philadelphia: John Benjamins, 27–54.

[ref3] Bott L. Rees A. Frisson S. (2016). The time course of familiar metonymy. J. Exp. Psychol. Learn. Mem. Cogn. 42, 1160–1170. doi: 10.1037/xlm0000218, PMID: 26618912

[ref4] Brdar-Szabó R. Brdar M. (2011). “What do metonymic chains reveal about the nature of metonymy?” in Defining Metonymy in Cognitive Linguistics: Towards a Consensus View. eds. Benczes R. Barcelona A. Ruiz de Mendoza F. J. (Amsterdam & Philadelphia: John Benjamins), 217–248.

[ref5] Cacciari C. Glucksberg S. (1994). “Understanding figurative language,” in Handbook of Psycholinguistics. ed. Gernsbacher M. A. (San Diego, CA: Academic Press), 447–477.

[ref6] Chen X. Li F. (2019). The length of preceding context influences metonymy processing: Evidences from eye tracking experiment. Rev. Cogn. Linguist. 17, 243–256.

[ref7] Cronk B. Lima S. Schweigert W. (1993). Idioms in sentences: effects of frequency, literalness, and familiarity. J. Psycholinguist. Res. 22, 59–82. doi: 10.1007/BF01068157

[ref8] Duffy S. Rayner K. (1990). Eye movements and anaphor resolution: effects of antecedent typicality and distance. Lang. Speech 33, 103–119. doi: 10.1177/002383099003300201, PMID: 2283922

[ref9] Estill R. Kemper S. (1982). Interpreting idioms. J. Psycholinguist. Res. 11, 559–568. doi: 10.1007/BF01067612

[ref10] Filik R. Hunter C. M. Leuthold H. (2015). When language gets emotional: irony and the embodiment of affect in discourse. Acta Psychol. 156, 114–125. doi: 10.1016/j.actpsy.2014.08.007, PMID: 25213155

[ref11] Frisson S. (2009). Semantic underspecification in language processing. Lang. Ling. Compass 3, 111–127. doi: 10.1111/j.1749-818X.2008.00104.x

[ref12] Frisson S. Pickering M. (1999). The processing of metonymy: evidence from eye movements. J. Exp. Psychol. Learn. Mem. Cogn. 25, 1366–1383. PMID: 10605827 10.1037//0278-7393.25.6.1366

[ref13] Frisson S. Pickering M. (2001). Obtaining a figurative interpretation of a word: support for underspecification. Metaphor. Symb. 16, 149–171. doi: 10.1080/10926488.2001.9678893

[ref14] Frisson S. Pickering M. (2007). The processing of familiar and novel senses of a word: why reading Dickens is easy but reading Needham can be hard. Lang. Cogn. Process. 22, 595–613. doi: 10.1080/01690960601017013

[ref15] Gerrig R. (1989). The time course of sense creation. Mem. Cogn. 17, 194–207. doi: 10.3758/BF03197069, PMID: 2927317

[ref16] Gerring R. Healy A. (1983). Dual processes in metaphor understanding: comprehension and appreciation. J. Exp. Psychol. Learn. Mem. Cogn. 9, 667–675. doi: 10.1037/0278-7393.9.4.667

[ref17] Gibbs R. W. (1980). Spilling the beans on understanding and memory for idioms in conversation. Mem. Cogn. 8, 149–156. doi: 10.3758/BF03213418, PMID: 7382816

[ref18] Gibbs R. W. (1986). Skating on thin ice: literal meaning and understanding idioms in conversation. Discourse Process. 9, 17–30. doi: 10.1080/01638538609544629

[ref19] Gibbs R. W. (1990). Comprehending figurative referential descriptions. J. Exp. Psychol. Learn. Mem. Cogn. 16, 56–66. doi: 10.1037/0278-7393.16.1.56, PMID: 1688454

[ref20] Gibbs R. W. (2006a). Embodiment in Cognitive Science. New York: Cambridge University Press.

[ref21] Gibbs R. W. (2006b). Metaphor interpretation as embodied simulation. Mind Lang. 21, 434–458. doi: 10.1111/j.1468-0017.2006.00285.x

[ref22] Gibbs R. W. Gonzales G. P. (1985). Syntactic frozenness in processing and remembering idioms. Cognition 20, 243–259. doi: 10.1016/0010-0277(85)90010-1, PMID: 4064608

[ref23] Glucksberg S. (1991). Beyond literal meanings: The psychology of allusion. Psychol. Sci. 2, 146–152. doi: 10.1111/j.1467-9280.1991.tb00122.x

[ref24] Goossens L. (1990). Metaphtonymy: The interaction of metaphor and metonymy in expressions for linguistic action. Cogn. Linguist. 1, 323–342. doi: 10.1515/cogl.1990.1.3.323

[ref25] Grice H. P. (1975). “Logic and conversation,” in Syntax and Semantics. *vol. 3.* eds. Cole P. Morgan J., (New York: Academic Press), 41–58.

[ref26] Grice H. P. (1989). Studies in the Way of Words. Cambridge, MA: Harvard University Press.

[ref27] Henderson J. M. Pollatsek A. Rayner K. (1989). Covert visual attention and extrafoveal information use during object identification. Percept. Psychophys. 45, 196–208. doi: 10.3758/BF03210697, PMID: 2710617

[ref28] Inhoff A. W. Lima S. D. Carroll P. J. (1984). Contextual effects on metaphor comprehension in reading. Mem. Cogn. 12, 558–567. doi: 10.3758/BF03213344, PMID: 6085393

[ref29] Janus R. A. Bever T. G. (1985). Processing of metaphoric language: An investigation of the three-stage model of metaphor comprehension. J. Psycholinguist. Res. 14, 473–487. doi: 10.1007/BF01666722

[ref30] Jodłowiec M. Piskorska A. (2015). Metonymy revisited: towards a new relevance-theoretic account. Intercult. Pragmat. 12, 161–187. doi: 10.1515/ip-2015-0009

[ref31] Keysar B. (1989). On the functional equivalence of literal and metaphorical interpretations in discourse. J. Mem. Lang. 28, 375–385. doi: 10.1016/0749-596X(89)90017-X

[ref32] Lakoff G. Johnson M. (1999). Philosophy in the Flesh: The Embodied Mind and its Challenge to Western Thought. New York: Basic Books.

[ref33] Littlemore J. (2015). Metonymy. Hidden Shortcuts in Language, Thought, and Communication. Cambridge: Cambridge University Press.

[ref34] Lowder M. W. Gordon P. C. (2013). It’s hard to offend the college: effects of sentence structure on figurative-language processing. J. Exp. Psychol. Learn. Mem. Cogn. 39, 993–1011. doi: 10.1037/a0031671, PMID: 23421507 PMC3714341

[ref36] McElree B. Murphy G. L. Ochoa T. (2006). Time course of retrieving conceptual information: A speed-accuracy trade-off study. Psychon. Bull. Rev. 13, 848–853. doi: 10.3758/BF03194008, PMID: 17328384 PMC2323592

[ref37] Myers J. L. Shinjo M. Duffy S. A. (1987). Degree of causal relatedness and memory. J. Mem. Lang. 26, 453–465. doi: 10.1016/0749-596X(87)90101-X

[ref38] Onishi K. H. Murphy G. L. (1993). Metaphoric reference: when metaphors are not understood as easily as literal expressions. Mem. Cogn. 21, 763–772. doi: 10.3758/BF03202744 8289654

[ref39] Ortony A. Schallert D. L. Reynolds R. E. Antos S. J. (1978). Interpreting metaphors and idioms: Some effects of context on comprehension. J. Verbal Learn. Verbal Behav. 17, 465–477. doi: 10.1016/S0022-5371(78)90283-9

[ref40] Paivio A. (1979). “Psychological processes in the comprehension of metaphor,” in Metaphor and Thought. ed. Ortony A. (Cambridge, England: Cambridge University Press), 150–171.

[ref41] Panther K.-U. (2005). “The role of conceptual metonymy in meaning construction,” in Cognitive Linguistics. Internal Dynamics and Interdisciplinary Interaction. eds. Ruiz de Mendoza F. J. Peña S. (Berlin & New York: Mouton de Gruyter), 353–386.

[ref42] Panther K.-U. Thornburg L. (2018). “What kind of reasoning mode is metonymy?” in Conceptual Metonymy. Methodological, Theoretical, and Descriptive Issues. eds. Blanco Carrión O. Barcelona A. Pannain R. (Amsterdam & Philadelphia: John Benjamins), 121–160.

[ref43] Pickering M. J. Traxler M. J. (1998). Plausibility and recovery from garden paths: An eye-tracking study. J. Exp. Psychol. Learn. Mem. Cogn. 24, 940–961. doi: 10.1037/0278-7393.24.4.940

[ref44] Piñango M. M. Zhang M. Foster-Hanson E. Negishi M. Lacadie C. Constable R. T. (2017). Metonymy as referential dependency: psycholinguistic and neurolinguistic arguments for a unified linguistic treatment. Cogn. Sci. 41, 351–378. doi: 10.1111/cogs.12341, PMID: 26887916

[ref45] Ruiz de Mendoza F. J. (2017). “Metaphor and other cognitive operations in interaction: From basicity to complexity,” in Metaphor: Embodied Cognition, and Discourse. ed. Hampe B. (Cambridge: Cambridge University Press), 138–159.

[ref46] Ruiz de Mendoza Ibáñez F. (2021). “Conceptual metonymy theory revisited. Some definitional and taxonomic issues,” in The Routledge Handbook of Cognitive Linguistics. eds. Wen X. Taylor J. (London and New York: Routledge), 204–227.

[ref47] Ruiz de Mendoza Ibáñez F. Galera A. (2014). Cognitive Modeling. A Linguistic Perspective. Amsterdam & Philadelphia: John Benjamins.

[ref48] Ruiz de Mendoza Ibáñez F. Pérez Hernández L. (2001). Metonymy and the grammar: motivation, constraints and interaction. Lang. Commun. 21, 321–357. doi: 10.1016/S0271-5309(01)00008-8

[ref49] Schraw G. (1995). Components of metaphoric processing. J. Psycholinguist. Res. 24, 23–38. doi: 10.1007/BF02146098

[ref50] Searle J. R. (1979). Expression and Meaning: Studies in the Theory of Speech Acts. United Kingdom: Cambridge University Press.

[ref51] Slabakova R. Cabrelli Amaro J. Kang S. K. (2013). Regular and novel metonymy in native Korean, Spanish, and English: experimental evidence for various acceptability. Metaphor. Symb. 28, 275–293. doi: 10.1080/10926488.2013.826556

[ref001] Steen G. J. (2007). Finding metaphor in discourse: pragglejaz and beyond. Cult. Lang. Represent. 5, 9–25.

[ref52] Wilson D. Carston R. (2007). “A unitary approach to lexical pragmatics: relevance, inference and ad hoc concepts,” in Pragmatics. ed. Burton-Roberts N. (Basingstoke: Palgrave Macmillan), 230–259.

[ref53] Zarcone A. Padó S. Lenci A. (2014). Logical metonymy resolution in a words-as-cues framework: evidence from self-paced reading and probe recognition. Cogn. Sci. 38, 973–996. doi: 10.1111/cogs.12108, PMID: 24628505

[ref54] Zibin A. (2021). Blood metaphors and metonymies in Jordanian Arabic and English. Rev. Cogn. Linguist. 19, 26–50. doi: 10.1075/rcl.00075.zib

